# Machine Learning Decomposition of the Anatomy of Neuropsychological Deficit in Alzheimer’s Disease and Mild Cognitive Impairment

**DOI:** 10.3389/fnagi.2022.854733

**Published:** 2022-05-03

**Authors:** Ningxin Dong, Changyong Fu, Renren Li, Wei Zhang, Meng Liu, Weixin Xiao, Hugh M. Taylor, Peter J. Nicholas, Onur Tanglay, Isabella M. Young, Karol Z. Osipowicz, Michael E. Sughrue, Stephane P. Doyen, Yunxia Li

**Affiliations:** ^1^Department of Medical Imaging, Tongji Hospital, School of Medicine, Tongji University, Shanghai, China; ^2^Department of Neurology, Tongji Hospital, School of Medicine, Tongji University, Shanghai, China; ^3^Omniscient Neurotechnology, Sydney, NSW, Australia; ^4^International Joint Research Center on Precision Brain Medicine, XD Group Hospital, Xi’an, China

**Keywords:** cognitive impairment, neuropsychological tests, MRI, neural networks, machine learning, neuroimaging markers

## Abstract

**Objective:**

Alzheimer’s Disease (AD) is a progressive condition characterized by cognitive decline. AD is often preceded by mild cognitive impairment (MCI), though the diagnosis of both conditions remains a challenge. Early diagnosis of AD, and prediction of MCI progression require data-driven approaches to improve patient selection for treatment. We used a machine learning tool to predict performance in neuropsychological tests in AD and MCI based on functional connectivity using a whole-brain connectome, in an attempt to identify network substrates of cognitive deficits in AD.

**Methods:**

Neuropsychological tests, baseline anatomical T1 magnetic resonance imaging (MRI), resting-state functional MRI, and diffusion weighted imaging scans were obtained from 149 MCI, and 85 AD patients; and 140 cognitively unimpaired geriatric participants. A novel machine learning tool, Hollow Tree Super (HoTS) was utilized to extract feature importance from each machine learning model to identify brain regions that were associated with deficit and absence of deficit for 11 neuropsychological tests.

**Results:**

11 models attained an area under the receiver operating curve (AUC-ROC) greater than 0.65, while five models had an AUC-ROC ≥ 0.7. 20 parcels of the Human Connectome Project Multimodal Parcelation Atlas matched to poor performance in at least two neuropsychological tests, while 14 parcels were associated with good performance in at least two tests. At a network level, most parcels predictive of both presence and absence of deficit were affiliated with the Central Executive Network, Default Mode Network, and the Sensorimotor Networks. Segregating predictors by the cognitive domain associated with each test revealed areas of coherent overlap between cognitive domains, with the parcels providing possible markers to screen for cognitive impairment.

**Conclusion:**

Approaches such as ours which incorporate whole-brain functional connectivity and harness feature importance in machine learning models may aid in identifying diagnostic and therapeutic targets in AD.

## Introduction

Alzheimer’s Disease (AD) is the most common form of dementia, affecting millions worldwide (Prince et al., 2013). AD is characterized by the deposition of β-amyloid and tau protein, which often precede the onset of dementia symptoms by at least 10–20 years (Villemagne et al., 2013). AD initially progresses through a prodromal stage of mild cognitive impairment (MCI), defined as impairment in any single cognitive domain (Vega and Newhouse, 2014). Patients with MCI may also progress to other types of dementia, remain stable, or return to a cognitively unimpaired state (Giorgio et al., 2020). There is therefore a need to disentangle higher cognitive functioning in the neurodegenerative disease states and examine overlaps and differences to provide insight into both pathological and normal age-related neurocognitive functioning. This will in turn improve diagnostic and therapeutic approaches.

Currently the diagnosis of MCI and AD is based on clinical evaluation, while structural changes are often not detected in early disease, even when imaging is interpreted by experienced radiologists. Due to the advancement of machine learning algorithms and new data, we have the opportunity to analyze large data sets and build prediction models to inform clinical practice (Panch et al., 2018; Davenport and Kalakota, 2019). Various studies have utilized machine learning to predict the conversion of MCI to AD based on neuropsychological measures and clinical markers, with several studies focusing on neuroimaging models (Huang et al., 2020; Stamate et al., 2020; Syaifullah et al., 2020; Grueso and Viejo-Sobera, 2021). Wee et al. (2012) employed support vector machines to demonstrate that a multimodal approach combining functional and structural connectivity data improved the accuracy of MCI classification. Shi and Liu extracted features from resting-state functional magnetic resonance imaging (rsfMRI) to classify stages of MCI (Shi and Liu, 2020). Syaifullah et al. (2020) developed a technique combining SVM with voxel-based morphometry and MMSE scores which substantially outperformed radiologists in diagnosing AD. Jitsuishi and Yamaguchi investigated multiple types of modalities to demonstrate that diffusion parameters was most accurate in distinguishing early and late MCI (Jitsuishi and Yamaguchi, 2022). In contrast, most of the existing literature preselects features to focus the machine learning model’s classification. While this results in high diagnostic accuracy, potentially crucial information about the disease process may be discarded. In the case of resting state functional connectivity, several studies have demonstrated key changes in brain network architecture across several large-scale networks, including the default mode, salience and limbic networks (Prasad et al., 2013; Badhwar et al., 2017; Ye et al., 2019). Many of these changes are can also be seen up to 4 years before the symptomatic onset of AD (Wisch et al., 2020). Since these large-scale functional networks comprising the seven-network model detailed by Yeo et al. (2011) are responsible for the complex processes underlying cognition (Yeo et al., 2011), it is important to formulate a network-based model of cognition in AD. This feat is, however, complicated by the magnitude of the data, which requires sophisticated machine learning tools to both make predictions, but also identify clinically meaningful targets for treatment. Therefore, models incorporating whole-brain functional connectivity and employing a network-based analysis may provide actionable insight and guide symptom-specific therapies.

In this study, we performed functional connectivity-based analysis and utilized a recently described machine learning approach (Doyen et al., 2021) to identify commonalities and differences in brain regions across neuropsychological domains in a cohort of MCI and AD patients, and age-matched cognitively unimpaired subjects. We sought to explore patterns among these regions to identify potential markers which may be used in future studies to develop better disease classifiers. We believe our methods will provide a basis for the utility of functional connectivity in improving diagnosis and patient selection for treatment in MCI and AD.

## Materials and Methods

### Patient Cohort

Participants were recruited from the Department of Neurology and Memory Clinic in Shanghai Tongji Hospital between September 2017 and January 2021. All participants were Chinese, right-handed, and between 50 and 85 years old. The participants were divided into three groups: AD group, MCI and control group.

Exclusion criteria included: (1) definite history of stroke; (2) definite history of other diseases of the central nervous system such as infection, demyelinating diseases, and Parkinson’s disease; (3) definite history of mental illness such as schizophrenia, major depressive disorder; (4) serious physical disease; (5) alcohol or drug addiction; (6) unable to cooperate with neuropsychological tests; (7) MRI contraindication; (8) iodine allergy.

Subjects included in the AD group had to meet the clinical diagnostic criteria set out by the National Institute on Aging and the Alzheimer’s Association (NIA-AA) (Albert et al., 2011). Inclusion in the MCI group was based on the neuropsychological Jak/Bondi criteria (Bondi et al., 2014) and the Petersen/Winblad criteria as operationalized by the Alzheimer’s Disease Neuroimaging Initiative (ADNI) (Marder, 2005). Specifically, the criteria included (a) the subject and their caregiver had complaint of memory/cognitive decline; (b) Mini-Mental State Examination (MMSE) MMSE or MoCA-B scores met the following criteria adjusted by education: MMSE ≤ 24 for Junior high school or above, or MMSE ≤ 20 for primary school, or MMSE ≤ 17 for illiteracy; or MoCA-B ≤ 24 for Bachelor degree or above, or MoCA-B ≤ 22 for middle school, or MoCA-B ≤ 19 for primary school or below; (c) Clinical Dementia Rating Scale (CDR) of 0.5; (d) met any of the following three criteria: (1) at least two performances within a cognitive domain fell below the established cutoff (> 1SD); (2) at least two cognitive domains were impaired (> 1SD); (3) had more than one function described in instrumental activities of daily living (IADL-14) scale scored 0 point.

All participants provided informed consent. This study was reviewed and approved by the Institutional Review Board at Tongji University.

### Neuropsychological Testing

All participants underwent a comprehensive Neuropsychological Test Battery (NTB) that included the MMSE, MoCA-B, tCDR, IADL-14, and the Hachinski Ischemic Score (HIS). Memory function was assessed by the Hopkins Verbal Learning Test-Revised (HVLT-R, including immediate recall test, the 5-min delayed recall, the 20-min delayed recall test), and the logical memory test (Wechsler memory scale). Language function was measured by the Verbal Fluency test and the Boston Naming Test (BNT; the 30-item version). Executive function was assessed using the Shape Trail Test-A and B (STT-A, STT-B). The larger scores in STT-A or STT-B test indicates longer time to complete the task and poorer executive performance. Visual space navigation function was measured by the Rey-Osterrieth Complex Figure Test (CFT, including the copy test and the recall test). The assessments were performed by a neurology clinician qualified in neuropsychological assessment.

### Statistical Analysis

Differences in demographic factors and neuropsychological test scores were analyzed using non-parametric tests; the Kruskal–Wallis test for continuous data, and Fisher’s Exact test for categorical data.

### Imaging Protocol

All examinations were performed with a 3.0T MR system (Magneton Verio, Siemens Medical Systems, Erlangen, Germany) with an orthonormal head coil. During the MRI scan, all participants were asked to remain still in the supine position with the surrounding space being filled with sponge.

We here relied on diffusion weighted images (DWI) and rsfMRI data with the following parameters: DWI: b1 = 0 s/mm^2^, b2 = 1,000 s/mm^2^, b3 = 2,000 s/mm^2^/64 directions, Matrix = 112 × 112, FOV = 224 mm × 224 mm, TR = 2,400 ms, TE = 71 ms, 76 slices, 2 mm thickness, no gap. and rsfMRI: TR = 500 ms, TE = 30 ms, flip angle (FA) = 60°, FOV = 224 mm × 224 mm, matrix = 64 × 64, slices = 35, thickness = 3.5 mm, gap = 0.5 mm. A 3D MPRAGE (magnetization prepared rapid acquisition gradient echo) image (voxel size 1 mm × 1 mm × 1 mm, TE: 2.98 ms, TR: 2,530 ms, flip angle = 7°) was also obtained.

#### Diffusion Tractography Preprocessing Steps

The DWI images were processed using the Infinitome software (Omniscient Neurotechnology, 2020), which employs standard processing steps in the Python language. The processing pipeline includes the following: (1) the diffusion image is resliced to ensure isotropic voxels, (2) motion correction is performed using a rigid body registration algorithm to a baseline scan, (2) slices with excess movement (defined as DVARS > 2 sigma from the mean slice) are eliminated, (3) the T1 image is skull stripped using the HD-BET software (Isensee et al., 2019), which is inverted and aligned to the DWI image using a rigid alignment, which is then used as a mask to skull strip the aligned DWI image, (4) gradient distortion correction is performed by applying a diffeomorphic warping registration method between the DWI and T1 images, (5) The fiber response function is estimated and the diffusion tensors are calculated using constrained spherical deconvolution, (7) deterministic tractography is performed with uniform random seeding, 4 seeds per voxel, usually creating about 300,000 streamlines per brain.

#### Creation of a Personalized Brain Atlas Using Machine Learning Based Parcelation

**FIGURE 1:**
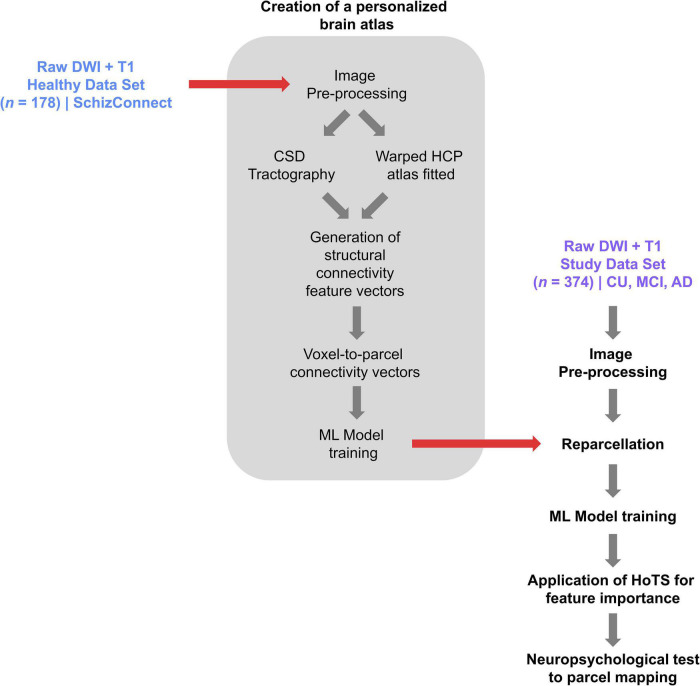
A flowchart demonstrating the process of creating a personalized atlas for each individual in the study sample, referred to as reparcelation.

In order to minimize the effects of gyral variation, we used a machine-learning based, subject specific version of the Human Connectome Project Multimodal Parcelation (HCP-MMP1) atlas (Glasser et al., 2016) generated based on each subject’s structural connectivity, which has been described elsewhere (Doyen et al., 2022). Figure 1 demonstrates the steps in creation of this personalized atlas. Briefly, a machine learning model was trained by entering preprocessed tractography data from 178 healthy controls obtained from the SchizConnect database to learn the structural connectivity pattern between voxels included within the 379 parcels of the HCP-MMP1 atlas. The same unaltered atlas was then warped onto each brain of the study sample. The trained machine learning model was then applied to each individual in the study sample to appoint voxels located at the endpoint of tractography streamlines to their most likely warped HCP parcelation based on the structural connectivity feature vectors, resulting in reparcelation of voxels. This method creates a version of the HCP-MMP1 atlas with 180 cortical parcels and 9 subcortical structures per hemisphere, along with the brainstem as one parcel.

We mapped the identified parcels to known resting-state networks based on the hierarchical structure described by Akiki and Abdallah (2019): the Default Mode Network (DMN), the Central Executive Network (CEN), Dorsal Attention Network (DAN), Salience Network (SN), Sensorimotor Network (SMN), and Visual Network (VN). These networks are based on the model first demonstrated by Yeo et al. (2011), who mapped specific parts of the cortex to known large-scale networks (Yeo et al., 2011). These networks have since been investigated across the spectrum of healthy and pathological cognitive states and have been demonstrated to underlie key cognitive processes. This template was therefore applied to our dataset to investigate patterns in the identified brain regions, and the networks with which they are associated.

#### rsfMRI Preprocessing Steps

The rsfMRI images were processed using standard processing steps including: (1) motion correction on the T1 and BOLD images using a rigid body alignment, (2) elimination of slices with excess movement (defined as DVARS > 2 sigma from the mean slice), (3) skull stripping of the T1 image using a convolutional neural net (CNN), which is inverted and aligned to the resting state bold image using a rigid alignment, and used as a mask to skull strip the rsfMRI image, (4) slice timing correction, (5) Global intensity normalization, (6) gradient distortion correction using a diffeomorphic warping method to register the rsfMRI and T1 images, (7) High variance confounds are calculated using the CompCor method (Behzadi et al., 2007); these confounds as well as motion confounds are regressed out of the rsfMRI image, and the linear and quadratic signals are detrended. Note this method does not perform global signal regression, (8) spatial smoothing is performed using a 4 mm FWHM Gaussian kernel. The personalized atlas created in previous steps is registered to the T1 image, and gray matter atlas regions are aligned with the gray matter regions in each subject’s scans. Thus, it is ideally positioned for extracting a BOLD time series, averaged over all voxels within a region, from all 379 regions (180 parcels from two hemispheres, plus 19 subcortical structures). The Pearson correlation coefficient is calculated between the BOLD signals of each unique area pair (self to self-inclusive), which yields 143,641 correlations.

#### Mapping of Neuropsychiatric Tests to Brain Regions Using the Hollow-Tree Super Method

We subsequently built machine learning models to predict a subject’s performance on a given test based on their functional connectome. The black box problem in machine learning generally limits the ability to utilize machine learning techniques in clinical practice, as there is generally a need-to-know which parts of the brain contribute to a given pathology. In order to address this, we used a boosted trees approach, called Hollow-tree Super (HoTS) (Doyen et al., 2021), to determine which features of each machine learning model, in this case the functional connectivity among brain regions, were contributing most to the model’s prediction of performance on each neuropsychological test. Performance in each test was classified by a tertile split, with the upper and lower tertiles classed as poor and good performance, respectively. The binarization of these test scores was necessary to apply logistic regression to continuous scores. However, since the mapping of psychometric testing to brain regions using machine learning is a novel technique, the thresholds were chosen to reflect differences between the worst and best performing groups. This was done to ensure that the model was identifying biological differences which were reflective of the clear functional differences between these two groups. Test specific tertile limits were: MOCA-B (< 17, poor performance; > 24 good performance), the Clock Drawing Test (< 3, poor performance; 3, good performance), and the Hopkins Verbal Learning Test Delayed Recall (0, poor performance; > 6, good performance). The prediction performance of each model was measured by the area under the receiver-operator curve (AUC-ROC). The binarization of each test and the class balances are provided in the Supplementary Figures.

## Results

### Patient Characteristics

Table 1 shows the demographic characteristics and median neuropsychological test scores, along with significance of differences reported on Kruskal–Wallis and Fisher’s Exact Tests. Across groups, there were significant differences in age and education between control, MCI and AD subjects, with control subjects being generally younger (median ± IQR, 71 ± 10 in control; 72 ± 11 in MCI; and 75 ± 10 in AD) and having attained a greater number of years of education (12 ± 6 in control; 9 ± 3 in MCI; 9 ± 3 in AD). Additionally, there were significant group differences in all neuropsychological tests conducted, with MCI and AD subjects performing worse.

**TABLE 1:** Subject demographics.

	Control (*n* = 140) Median (IQR)	MCI (*n* = 149) Median (IQR)	AD (*n* = 85) Median (IQR)	*p*-value
Age (years)	71 (65, 75)	72 (70, 81)	75 (67, 77)	<0.001
Education (years)	12 (9, 15)	9 (9, 12)	9 (9, 12)	<0.001
Sex *n* (%) Female Male Missing	68 (48.6) 72 (51.4) 0	81 (54.7) 67 (45.3) 1	53 (62.4) 32 (37.6) 0	0.130
MMSE	27.5 (26, 28)	24 (22, 26)	15 (11, 19)	<0.001
MOCA-B	24 (22, 26)	16 (14, 19)	8 (5, 11)	<0.001
Wechsler Memory Scale Logical Memory	9 (7, 11)	6 (4, 7.25)	2 (1, 4)	<0.001
Hopkins Verbal Learning Test (Immediate)	19 (16, 22)	14 (11, 17)	7 (3, 10)	<0.001
Hopkins Verbal Learning Test (Delayed 5 min)	7 (5, 8)	3 (0, 5)	0 (0, 0)	<0.001
Hopkins Verbal Learning Test (Delayed 20 min)	7 (5, 8)	3 (0, 5)	0 (0, 0)	<0.001
Boston Naming Test	24 (20, 26)	21 (17, 24)	15 (11.5, 20)	<0.001
Boston Naming Test Articulateness and Fluency	15 (12, 17)	12 (9, 14)	6 (4, 9)	<0.001
Rey Osterrieth Complex Figure Imitation	32 (6, 35)	21 (6, 33.5)	6 (1, 22)	<0.001
Rey Osterrieth Complex Figure Recall	11 (5, 19)	4 (0, 10)	0 (0, 0)	<0.001
Shape Trail Test Part A	56 (44, 73)	73 (58, 94.75)	108.5 (80.75, 140.5)	<0.001
Shape Trail Test Part B	145 (109, 179)	181 (153, 230)	232 (189.5, 273)	<0.001

*Non-parametric tests conducted, with median and interquartile range (IQR) reported. MCI, mild cognitive impairment; AD, Alzheimer’s Disease.*

### Machine Learning Reveals Parcels Underlying Cognitive Deficits

**FIGURE 2:**
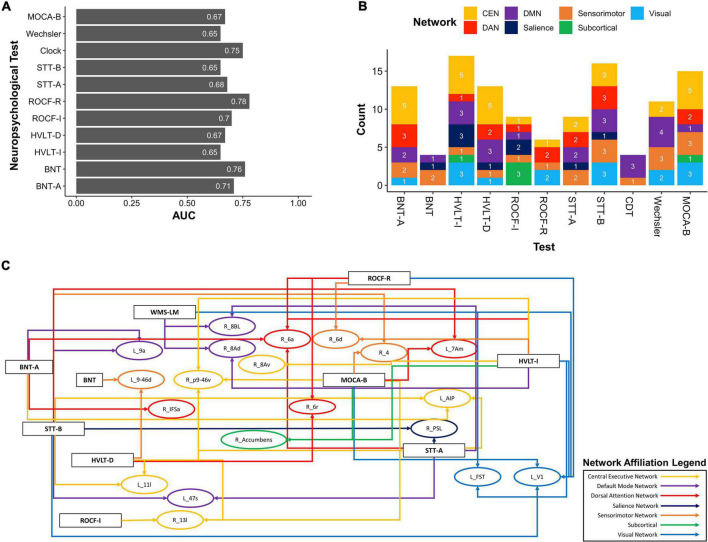
Baseline resting-state fMRI parcels predicting deficits in neuropsychological tests. **(A)** 11 neuropsychological tests surpassed an AUC of 0.65. **(B)** Parcel to network mapping showed that parcels in the CEN and DMN were most frequently associated with poor performance in neuropsychological testing, though other networks were also represented. **(C)** Neuropsychological test to parcel mapping of twenty parcels which were predictors of at least two tests. The parcels have been placed in relative anatomical positions, while the colors of the arrows represent the networks associated with the parcels. Each arrow is drawn from the neuropsychological test (rectangle) to a single parcel (oval). CEN, Central Executive Network; DAN, Dorsal Attention Network; DMN, Default Mode Network; BNT-A, Boston Naming Test—Articulateness and Fluency; BNT, Boston Naming Test; HVLT-I, Hopkins Verbal Learning Test—Immediate Memory; HVLT-D, Hopkins Verbal Learning Test—Delayed Recall; ROCF-I, Rey Osterrieth Complex Figure Imitation; ROCF-R, Rey Osterrieth Complex Figure Recall; STT-A, Shape Trail Test—Part A; STT-B, Shape Trail Test Part B.

Applying our HoTS methodology to determine features of the functional connectome associated with performance in each neuropsychological test, eleven models had a test AUC greater than 0.65 (Figure 2A): Boston Naming Test (BNT), Boston Naming Test—Articulateness and Fluency (BNT-A), Hopkins Verbal Learning Test Immediate Recall (HVLT-I), Hopkins Verbal Learning Test 5 min Delayed Recall (HVLT-D), Rey Osterrieth Complex Figure Imitation (ROCF-I), Rey Osterrieth Complex Figure Recall (ROCF-R), Shape Trail Test Part A (STT-A), Shape Trail Test Part B (STT-B), Wechsler Memory Scale Logical Memory (WMS-LM), Clock Drawing Test (CDT), and MOCA-B. Results were categorized into the domains of Language (BNT), Verbal Learning and Memory (HVLT-I, HVLT-D, WMS-LM), Attention and Executive Function (BNT-A, STT-A, STT-B), Visuospatial Function (ROCF-I, ROCF-R, CDT), and MOCA-B as a standalone general cognitive test.

### Injury to Sensory and Higher Order Association Regions Underlie Naming Deficits in AD

Right 3a (SMN), right 10r (DMN), left OP1 (SMN), and left 9–46 days (SN) were most predictive of poor performance in the BNT, whereas there were no predictors of absence of deficit. Supplementary Figure 1 shows the log odds of each parcel as a predictor for performance in the BNT.

### DLPFC and IFOF-Connected Regions Are Abnormal in People With Impaired Verbal Recall

Table 2 lists the parcels identified by each model for tests associated with verbal learning and memory. Poor performance in the HVLT-I was associated with multiple core networks, including DMN, CEN, SN, and VN. Anatomically, a subset of these parcels were in the right dorsolateral prefrontal cortex (DLPFC) (right 8Av, right 8Ad, right p9-46v), right premotor area (area 6d, area 6a, area 6r), right insula (right PoI2), left DLFPC (left 46), left medial frontal lobe (left 10r) and left occipital cortex (left V1, left V3B, left FST). The distribution of these parcels suggest that the model may be highlighting alterations in the connectivity of the inferior fronto-occipital fascicle (IFOF). Expectedly, absence of deficit in the HVLT-I was linked to regions associated with working memory: left PHA1, left p10p, and left 23 days.

**TABLE 2:** Predictors of performance in neuropsychiatric tests associated with verbal learning and memory.

Wechsler Memory Scale				Hopkins Verbal Learning Test Immediate Memory				Hopkins Verbal Learning Test Delayed Recall			
AUC = 0.65				AUC = 0.65				AUC = 0.67			
Deficit Present		Deficit Absent		Deficit Present		Deficit Absent		Deficit Present		Deficit Absent	
Area	Network	Area	Network	Area	Network	Area	Network	Area	Network	Area	Network
L_V1	Visual	L_PHA1	DMN	R_H	DMN	L_PHA1	DMN	L_9-46d	Salience	L_PIT	Visual
R_TE1p	CEN	R_TF	DMN	R_23d	CEN	L_p10p	CEN	R_LIPd	CEN	L_23c	Salience
R_6d	SMN	R_AAIC	CEN	R_8Av	CEN	L_23d	CEN	R_6r	DAN	L_9m	DMN
R_47m	DMN	R_9a	DMN	R_6a	DAN			L_11l	CEN	R_TPOJ1	DMN
L_FST	Visual	L_MI	Salience	R_pOFC	CEN			R_7AL	SM	R_55b	Salience
R_8BL	DMN	L_RSC	CEN	R_6d	SM			R_45	DMN	R_LO2	Visual
R_11l	CEN	R_PHA1	DMN	R_Accumbens	SC			L_10pp	DMN	L_VMV2	Visual
L_1	SMN	L_23d	CEN	L_FST	Visual			L_PEF	DAN		
L_3b	SMN	R_Amygdala	SC	L_V1	Visual			R_PGs	CEN		
R_STSva	DMN			R_8Ad	DMN			R_13l	CEN		
R_8Ad	DMN			L_IFJp	CEN			R_V4t	Visual		
				L_SCEF	Salience			R_p9-46v	CEN		
				L_V3B	Visual			L_p32	DMN		
				R_PoI1	Salience						
				L_10r	DMN						
				R_p9-46v	CEN						
				L_46	Salience						

*CEN, Central Executive Network; DAN, Dorsal Attention Network; DMN, Default Mode Network; SC, Subcortical Deficit Present refers to good performance, whereas Deficit Absent refers to poor performance in a given test, as defined in the methodology.*

### Deficits in Delayed and Episodic Verbal Recall Highlight Possible Functional Compensation

The CEN and DMN were most associated with deficits in the HVLT-D, whereas the VN, SN, and DMN were linked to absence of deficits. Notably, the right-sided analogs of speech areas, right 55b, and right 45 were associated with absence and presence of deficit, respectively.

When looking at the substrates for verbal episodic memory, presence of deficit in the WMS-LM was associated with the DMN, CEN, SMN and VN; while absence of deficit was associated with parcels in the medial temporal lobe and limbic components of the DMN, CEN, SN and the right Amygdala. Supplementary Figures 2–4 show the log odds of each parcel as a predictor for performance in each language test.

### Performance in Executive Function Is Associated With Executive and Sensory Networks

The parcels our models associated with performance in tests associated with attention and executive function are listed in Table 3. For the BNT-A, parcels predictive of deficit were affiliated with the CEN, DMN, DAN, and SMN. Conversely, parcels associated with absence of deficit were mostly associated with the DMN, especially its limbic components.

**TABLE 3:** Predictors of performance in neuropsychiatric tests associated with attention and executive function.

Boston naming test articulateness and fluency				Shape trail test (Part A)				Shape trail test (Part B)			
AUC = 0.71				AUC = 0.68				AUC = 0.65			
Deficit Present		Deficit Absent		Deficit Present		Deficit Absent		Deficit Present		Deficit Absent	
Area	Network	Area	Network	Area	Network	Area	Network	Area	Network	Area	Network
R_IFSa	DAN	R_IFJp	CEN	L_47s	DMN	L_IFJa	CEN	L_47s	DMN	R_FOP1	Salience
R_47s	DMN	L_PGs	DMN	L_13l	CEN	R_PH	Visual	L_11l	CEN	L_p32pr	Salience
L_9a	DMN	L_STGa	DMN	R_LBelt	SMN	R_PFt	DAN	L_V3CD	Visual	R_TE2p	DAN
R_2	SMN	R_OP2-3	SMN	L_AIP	DAN	L_p47r	CEN	R_a9-46v	CEN	R_43	SMN
L_6mp	SMN	L_PeEc	DMN	R_p9-46v	CEN	R_PHT	DAN	L_AIP	DAN		
L_LIPv	Visual	R_H	DMN	R_PSL	Salience	L_PFm	CEN	L_7Am	DAN		
R_6a	DAN			R_6a	DAN	R_PBelt	SMN	L_s6-8	CEN		
R_OFC	CEN			R_8BL	DMN	L_p24	CEN	R_STSdp	DMN		
L_IFSa	CEN			R_43	SMN	L_Pir	SMN	R_PSL	Salience		
L_d32	DMN					R_8BM	CEN	L_V1	Visual		
R_9-46d	CEN					R_RI	SMN	L_PFcm	SM		
L_AVI	CEN							L_PH	Visual		
L_AIP	DAN							L_9a	DMN		
								R_4	SMN		
								R_TA2	SMN		
								R_IFSa	DAN		

*CEN, Central Executive Network; DAN, Dorsal Attention Network; DMN, Default Mode Network; SC, Subcortical Deficit Present refers to good performance, whereas Deficit Absent refers to poor performance in a given test, as defined in the methodology.*

Furthermore, deficits in the STT-A were associated with several networks, including the DMN, CEN, DAN, and SMN, while absence of deficit was predicted by parcels in the CEN, and SMN. In the STT-B, the DAN, CEN, DMN, SMN, and VN were associated with deficit; whereas the SN, DAN and SMN were associated with absence of deficit. Both tests demonstrate impairment in areas known to be associated with executive function, though there was also involvement of unexpected networks such as the SMN. Supplementary Figures 5–7 detail the feature importance of each parcel as a predictor in the models for the attention and executive function tests.

### Visuospatial Deficits Are Associated With Multiple Large-Scale Networks

Table 4 shows the identified parcels associated with performance in visuospatial tests, along with their network affiliation. Presence of deficit in the ROCF-I was mainly associated with subcortical structures and SN; while absence of deficit was mostly linked to the VN and DMN. Functional connectivity of parcels in the DAN and VN were predictors of deficit in ROCF-R, whereas absence of deficit was linked to the limbic and language components of the DMN and CEN.

**TABLE 4:** Predictors of performance in neuropsychiatric tests associated with visuospatial function.

Rey osterrieth complex figure imitation				Rey osterrieth complex figure recall				Clock drawing test			
AUC = 0.70				AUC = 0.78				AUC = 0.75			
Deficit present		Deficit Absent		Deficit Present		Deficit absent		Deficit Present		Deficit absent	
Area	Network	Area	Network	Area	Network	Area	Network	Area	Network	Area	Network
R_Caudate	SC	L_TE1p	CEN	L_OFC	CEN	L_TGd	DMN	L_31a	DMN	L_PBelt	SMN
L_p32pr	Salience	L_SFL	DMN	R_6r	DAN	R_V1	Visual	L_STSva	DMN	R_TE1m	CEN
Brainstem	SC	R_PoI2	SMN	R_6a	DAN	L_TE1p	CEN	L_44	DMN	L_Thalamus	SC
L_Caudate	SC	L_PHA1	DMN	R_V8	Visual	L_STGa	DMN	R_5m	SMN	L_PGs	DMN
L_EC	DMN	R_PHT	DAN	R_6d	SMN	R_STSda	DMN			R_V8	Visual
R_13l	CEN	R_PH	Visual	L_V1	Visual	L_Accumbens	SC			R_IP2	CEN
L_PF	Salience	L_PHA3	DMN			L_TE1a	DMN			L_PHA1	DMN
L_5L	SMN	R_V2	Visual			L_a32pr	CEN			L_EC	DMN
R_PFt	DAN	L_VIP	Visual			R_TF	DMN			L_5m	SMN
		L_LIPv	Visual			R_OP1	SMN			R_9-46d	CEN
		L_8Av	DMN			L_MBelt	SMN			L_TE2p	DAN
						R_Thalamus	SC			L_IP1	CEN
						L_IFJa	CEN			R_PBelt	SMN
						L_PeEc	DMN			L_VVC	Visual
										R_FOP2	SMN
										R_Thalamus	SC

*CEN, Central Executive Network; DAN, Dorsal Attention Network; DMN, Default Mode Network; SC, Subcortical Deficit Present refers to good performance, whereas Deficit Absent refers to poor performance in a given test, as defined in the methodology.*

Moreover, language regions of the DMN and the SMN were associated with deficit in the CDT, whereas the SMN, CEN, and DMN were associated with absence of deficit.

Log odds of each parcel as a predictor of performance for the visuospatial tests are shown in Supplementary Figures 8–10.

### Parcels Associated With Performance in MOCA-B May Provide Markers of Cognitive Decline

Finally, poor performance in the MOCA-B was associated with the CEN (right 13l, left POS2, right 8Av, right p9-46v, left PFm), VN (left V1, right VIP, left V7), and SMN (right 4, right Pir, left 7PC); and absence of deficit was linked to the SMN (right FOP2, left MBelt), SN (left FOP1), DMN (right PCV) and VN (right PIT). Although the AUC of 0.68 for this model was relatively low, these areas may be studied further as possible markers to predict cognitive decline. The log odds for each parcel is provided in Supplementary Figure 11.

### Overlaps and Differences Across Cognitive Domains May Provide Insight Into Distinct Progression Trajectories in AD

**FIGURE 3:**
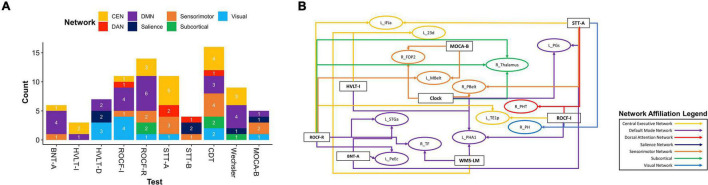
Baseline resting-state fMRI parcels predicting absence of deficits in neuropsychological tests. **(A)** Parcel to network mapping showed that parcels in the CEN and DMN were most frequently associated with absence of deficit in neuropsychological testing, and these networks were the most common overall. **(B)** Neuropsychological test to parcel mapping of fourteen parcels which were predictors of good performance in at least two tests. The parcels have been placed in relative anatomical positions, while the colors of the arrows represent the networks associated with the parcels. Each arrow is drawn from the neuropsychological test (rectangle) to a single parcel (oval). CEN, Central Executive Network; DAN, Dorsal Attention Network; DMN, Default Mode Network; BNT-A, Boston Naming Test—Articulateness and Fluency; BNT, Boston Naming Test; HVLT-I, Hopkins Verbal Learning Test—Immediate Memory; HVLT-D, Hopkins Verbal Learning Test—Delayed Recall; ROCF-I, Rey Osterrieth Complex Figure Imitation; ROCF-R, Rey Osterrieth Complex Figure Recall; STT-A, Shape Trail Test—Part A; STT-B, Shape Trail Test Part B.

We next explored overlaps between parcels associated with each neuropsychological test to derive patterns which may aid in diagnosis. At a network level, predictors of deficit in each test were affiliated with a variety of networks, although the CEN was most common, followed by the DMN (Figure 2B). We identified 20 parcels which were predictors of poor performance in at least two neuropsychological tests (Figure 2C). Most of these were affiliated with the CEN, DMN, and DAN. When examining parcels associated with absence of deficit, the DMN and CEN were also over-represented (Figure 3A). 14 parcels were predictors of good performance in at least two neuropsychological tests (Figure 3B), and most parcels were associated with temporal structures affiliated with the DMN.

**FIGURE 4:**
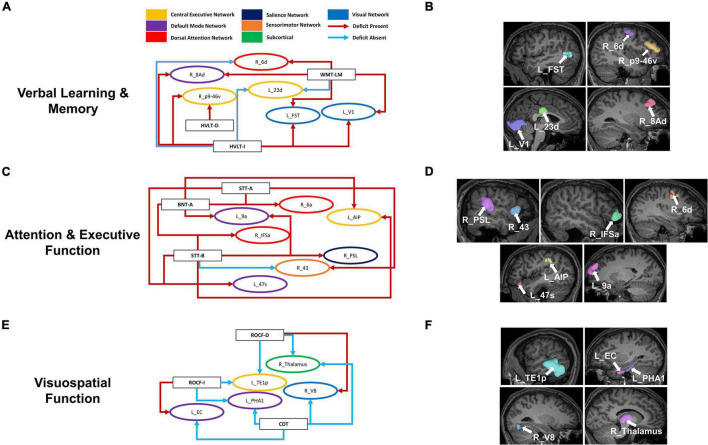
Overlaps within cognitive domains may provide insight into functional compensation and cognitive trajectories in AD. Parcels associated with performance in at least two neurocognitive tests associated within each cognitive domain: Verbal Learning and Memory **(A)**, Attention and Executive Function **(C)**, and Visuospatial Function **(E)**. Each arrow is drawn from the neuropsychological test (rectangle) to a single parcel (oval). The arrows are colored by whether the association was to poor performance (Deficit Present) or good performance (Deficit Absent) as defined in the methodology for each test, while the parcels are colored by their associated networks. Anatomical locations of the given parcels are also shown on a T1 MRI **(B,D,F)**. BNT-A, Boston Naming Test—Articulateness and Fluency; BNT, Boston Naming Test; HVLT-I, Hopkins Verbal Learning Test—Immediate Memory; HVLT-D, Hopkins Verbal Learning Test—Delayed Recall; ROCF-I, Rey Osterrieth Complex Figure Imitation; ROCF-R, Rey Osterrieth Complex Figure Recall; STT-A, Shape Trail Test—Part A; STT-B, Shape Trail Test Part B, WMS-LM, Wechsler Memory Scale—Logical Memory.

Although each neuropsychological test assesses distinct functions, to identify commonalities and differences in our data, we grouped parcels by the cognitive domain associated with each test. We then identified parcels which were predictors of performance in at least two neurocognitive tests within each domain. Language and the MOCA-B were left out of this analysis as only one model was associated with each of these. Within Verbal Learning and Memory, four out of six parcels were associated with poor performance in at least two tests, whereas right 6d was associated with poor performance in the WMS-LM and good performance in HVLT-I, and left 23d was associated with good performance in both the WMS-LM and HVLT-I (Figures 4A,B). Common parcels associated with attention and executive function were located within the frontal and parietal lobes (Figures 4C,D). All parcels were associated with poor performance, except right 43, which was associated with poor performance in the STT-A, but good performance in STT-B. Given the difference between the two tasks is the cognitive flexibility required in STT-B, which is the harder task, this region may be indicating initial functional compensation which is successful in some individuals. Finally, all common parcels associated with visuospatial function were in the temporooccipital region, with the addition of the right Thalamus (Figures 4E,F). All parcels identified were common predictors of absence of deficit, except right V8, which was associated with absence of deficit in the CDT and presence of deficit in the ROCF-D; and left EC, which was associated with absence of deficit in the CDT and presence of deficit in the ROCF-I. These differences may again be indicative of different stages or trajectories of disease, though the lack of longitudinal data limited further analysis.

**FIGURE 5:**
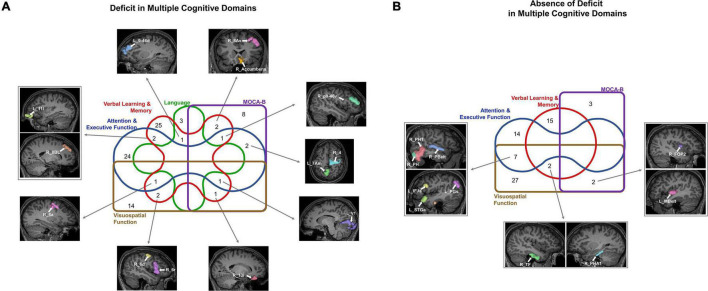
Commonalities across cognitive domains may provide potential targets for screening, diagnosis, and treatment. The Venn diagrams show the parcels which are predictors of **(A)** deficit and **(B)** absence of deficit across at least two cognitive domains.

We finally looked at commonalities between domains by classifying the 20 parcels associated with presence of deficit in two or more tests into five domains based on the associated neuropsychological test (Figure 5A). The common areas tended to be in the frontal lobe and affiliated with the CEN (right p9-46v, right 13l, right 8Av, left 11l), DAN (right 6a, right 6d) DMN (right 8BL, left 7Am), SMN (right 4, right 6a), SN (left 9–46d), and right Accumbens. Interestingly, we noted a convergence of verbal learning and visuospatial function at areas right 6a, right 6d and right 6r. Furthermore, a similar grouping of parcels associated with absence of deficit (Figure 5B) revealed several temporal and perisylvian regions within the DMN (R_TF, R_PHA1, left PGs, left STGa), SMN (left MBelt, right PBelt, right FOP2), DAN (right PHT), CEN (left IFJa) and VN (right PH).

## Discussion

Despite rising prevalence and ongoing efforts, we still lack adequate tools to track the progression of MCI and diagnose AD early in its pathological stage. Data-driven methods are needed to *a priori* identify pathological progression and functional decline and enable premorbid treatment. Our machine learning models demonstrate that different neurocognitive deficits in AD are associated with functional connectivity abnormalities in multiple bi-hemispheric neural networks. Notably, the limbic components of the DMN and CEN, and the DAN were most associated with performance measures. Deficits in multiple domains revealed impairments in top-down processing, and possible recruitment of analog areas in the contralateral hemisphere, which may underlie response to injury in AD. We propose these regions may serve as possible neuroimaging markers of disease progression, and aid in establishing clinical trajectories, though longitudinal data are needed to explore this further. Ultimately, we demonstrate the utility of data-driven methods to explore the neural networks underlying functional and cognitive deficits in AD. Further analyses have the potential to revolutionize diagnosis and treatment, and improve quality of life in AD.

### Cognitive Deficits in AD Arise From Impairment in Multiple Networks

A commonality between all the models in our analysis was the involvement of multiple networks in each cognitive task, and the involvement of areas which would not classically be associated with a given task. For example, deficits in naming were associated with regions outside of language or motor planning areas. Similarly, our data suggest that fluency and articulation deficits in AD may be caused by cognitive abnormalities in motor planning, with several premotor area parcels being associated with deficit. The same premotor regions were also associated with visuospatial deficits in our dataset. Previous studies have identified a possible role of the premotor area in articulation planning (Paulesu et al., 1993; Marvel and Desmond, 2012; Liao et al., 2014). This may suggest that fluency and articulation difficulties in AD stem from a phenomenon paralleling ideational apraxia, rather than exclusively explicit damage to language areas. Overall, these data suggest that many deficits in AD present a multi-network cognitive problem, rather than localized functional deficits. A multi-network perspective of functional deficits in AD must be assumed when describing the disease which should underlie the development of any therapeutic intervention.

### The Role of the DLPFC and IFOF in Working Memory

Several parcels within the DLPFC were associated with poor performance in the immediate recall portion of the HVLT. The DLPFC bilaterally is known to play an important role in verbal and spatial working memory (Sisi and Yixuan, 2018). In AD, impaired plasticity of the left DLPFC, measured as potentiation of cortical evoked activity has been correlated with working memory (Kumar et al., 2017). Increased activation of the right DLPFC on the other hand has also been associated with memory deficits in AD (Erk et al., 2011; Liang et al., 2011). Whether this is a maladaptive or compensatory mechanism remains unclear. Our model for poor performance in the HVLT-I predominantly highlighted the right DLPFC, which may indicate a similar maladaptive reorganization or nociferous compensation.

Furthermore, our machine learning model associated the functional connectivity of parcels in the left occipital and frontal lobes with impaired immediate recall. The IFOF is a white matter bundle which runs between these two regions, connecting the frontopolar, orbitofrontal and inferior frontal cortices to the occipital lobe (Conner et al., 2018). The role of the IFOF remains controversial. Several neuroimaging and neuromodulation studies have suggested a role in visual semantic processing (Moritz-Gasser et al., 2013; Almairac et al., 2015; Yordanova et al., 2017). However, it is still unclear how this pathway functionally differs from the parallel, indirect path in the ventral stream, anatomically described as the inferior longitudinal fascicle (ILF)/uncinate fasciculus (UF). Since the IFOF is connected to executive areas in the frontal lobe, it is possible that the IFOF is primarily involved in top-down processing of language, whereby the frontal lobe aids in visual semantic processing by biasing the visual system toward cognitively relevant goals. Indeed, planning spoken language has been shown to require attentional control (Roelofs and Piai, 2011). Our data may therefore be pointing to a similar paradigm, whereby individuals performing poorly on a verbal working memory task have left IFOF deficits. The model for deficits in HVLT-I also identified the right insula. This may be suggestive of ineffective compensation by the right IFOF, which also has subinsular components, however, further functional and lesion studies are required to investigate these hypotheses.

### Modulating Compensatory Plasticity in AD

Our models highlighted several possible compensatory mechanisms underlying deficits in MCI and AD, including the recruitment of alternate networks and contralateral analogous areas. Further exploring these changes in functional connectivity hold potential in targeting neuroplastic responses to injury in AD. Congruent to this, a recent study on a small cohort of patients with subjective cognitive decline (SCD), MCI, and AD demonstrated decrease in functional connectivity centrality measures in the somatomotor and visual networks in SCD patients (Wang et al., 2019). In contrast, AD patients showed an increase in centrality measures, and the authors proposed that as associative networks such as the DMN, CEN, and DAN were damaged, there was attempted compensation in the primary sensory networks. The frequent association of the sensorimotor and visual networks with presence of deficit within our data may therefore be explained by maladaptive recruitment of these networks in AD patients. These findings were echoed by another study demonstrating increased connectivity within the prefrontal, parietal and occipital lobes in AD, alongside decreased connectivity between the frontal and prefrontal lobes (Wang et al., 2007). This phenomenon of anterior-posterior functional connectivity dysfunction (Tao et al., 2020) may also be reflected in our data set, as many parcels which were predictors of dysfunction in multiple tests were in the parietal and frontal cortices. Moreover, the use of functional connectivity measures in the diagnosis and treatment of AD may enable precision medicine to improve patient outcomes. Functional connectivity may be a marker of cognitive reserve (Topiwala et al., 2019; Ewers et al., 2021), a hypothesis which may underpin the different clinical trajectories seen in AD (van Loenhoud et al., 2019). Indeed, patients with higher cognitive reserve were seen to have a more efficient functional connectome, suggesting they are better able to cope with progressing AD pathology (Weiler et al., 2018). Exploring the individual differences in the ability of the brain to adapt to network changes may enable harnessing methods to improve compensation in response to AD.

### Predicting MCI Conversion to AD

There have been several studies demonstrating the utility of machine learning tools in predicting MCI conversion to AD, and in diagnosing AD, with varying degrees of success (Syaifullah et al., 2020; Kumar et al., 2021). Each study utilizes a different combination of features in their models, ranging from clinical data, neuropsychological testing (Battista et al., 2017; Gupta and Kahali, 2020), behavioral and psychiatric data (Gill et al., 2020; Lo et al., 2020), and various imaging modalities (Jo et al., 2019). It is however unclear how early these models can be employed to screen individuals. The heterogeneity in clinical trajectories, and the likely need to implement treatment much earlier than the onset of symptoms require biological markers which indicate risk prior to decline in neuropsychological testing. This accounts for the popularity of incorporating imaging modalities into many of these models, as they provide a minimally invasive means of diagnosis independent of neuropsychological testing. The strength of the present study is the ability of our machine learning model to provide visibility into the features of the functional connectome associated with cognitive function. In fact, the neuroanatomical information provided by this method, specifically the areas of coherent overlap between domains as highlighted in Figure 5, and differences within, and across domains can be integrated with the neuropathological perspective in AD to build a progression model which can be used in screening and diagnosis. For example, Vogel et al. (2021) identified four trajectories in AD based on tau deposition. Rate of tau deposition has also been associated with functional connectivity in AD (Franzmeier et al., 2020) and functionally connected regions demonstrated shared levels of tau (Franzmeier et al., 2019), The close link between tau deposition and cognitive decline warrants further exploration of the role of functional connectivity in tau progression in AD. While some regions we identified overlapped with common areas of β-amyloid and tau deposition (Braak and Braak, 1991; Braak et al., 2011), it is not possible to apply pathological models without longitudinal data, and on a mixed cohort of AD and MCI patients. Nonetheless, our methods can enable an exploration of the differential effects of β-amyloid and tau deposition and provide means of predicting pathological progression *in vivo*.

### Machine Learning Models in AD

It is difficult to directly compare the performance of our model to models used in previous studies. While most studies utilize machine learning to classify patients into a diagnostic category, often by focusing the model on a feature within a given modality, our model classified individuals into test performance based on functional connectivity. This was performed in order to identify specific anatomical regions associated with cognitive test performance, rather than develop a diagnostic tool. Nonetheless, many studies utilizing machine learning have reported excellent performance, with accuracy, sensitivity and specificity at times exceeding 99% (Odusami et al., 2021), though a recent systematic review demonstrated a mean accuracy of 75.4% for support vector machines in predicting progression of MCI to AD (Grueso and Viejo-Sobera, 2021). However, a primary reason preventing the implementation of these models in clinical practice is their replicability, where the same models which have often overfitted to the study sample will underperform with independent data sets (Beam et al., 2020; Crowley et al., 2020). This in turn may lead to false conclusions and prevent the implementation of machine learning in settings where it may revolutionize diagnostics. It is therefore imperative that applied methodology is rigorous and validated using independent data sets.

The novelty of the machine learning technique used in this study lies in the ability to identify which features the model is relying the most on to make its prediction. This is generally difficult to do when a model has a large number of features as its input, as is the case with functional connectivity among 379 brain regions. Traditional methods would instead attempt to identify significant features prior to feeding these into a model, therefore biasing the model to focus solely on, for example, the default mode network. This however potentially removes global network information which may reveal key insights about the pathology. Our method therefore provides an embedded solution to directly analyze rsfMRI data, though further analysis techniques are necessary to identify markers from the large amount of data that results from this method.

## Limitations

As stated previously, machine learning models are currently unable to determine which feature of the functional connectivity of each parcel the model uses to predict the response. The models may be highlighting either reduced activation, indicating deficits in the underlying network, or alternatively, there may be increased activity due to greater reliance on networks which are compensating, albeit ineffectively. The former may be used as markers in diagnosis and screening, whereas the latter could potentially be targeted using TMS to strengthen networks and improve symptoms, though current evidence for the therapeutic value of TMS in AD remains unclear (Weiler et al., 2020). Beyond improving computational techniques, this problem could be addressed through longitudinal studies on large cohorts to track functional connectivity changes and connect this with clinical data and comparing these observations to neuropathological data.

Furthermore, the functional connectivity features were resolved from baseline resting-state fMRI rather than task-based fMRI. Consequently, the parcels identified by each model are not necessarily those activated when completing these tasks, but rather an association between changes in the functional connectome and the performance in each test. Finally, we did not stratify our models by diagnosis due to our limited sample size, instead favoring more stable models by opting in for classification based on best and worst performance in neuropsychiatric testing.

## Conclusion

Diagnosing and treating AD remains one of the foremost challenges of modern medicine. We present a model of MCI and AD which provides further insight into the anatomical correlates of neuropsychological dysfunction. We demonstrate that function and dysfunction in AD is mediated by the interaction of several networks, including the DMN and CEN but also sensorimotor and visual networks. While other machine learning methods have been applied to AD with comparable performance, our machine learning model is able to peek into the black box and explain the network components contributing to models of AD directly from a whole-brain connectome. This avoids the elimination of potentially key network information which accompanies feature selection in targeted studies looking at specific networks. Focusing on whole-brain network changes in AD could potentially lead to disentangling factors contributing to a disparity between functioning individuals and those with inefficient compensation or low cognitive reserve. In turn, these insights may empower therapeutic targeting to improve disease trajectory and identify early biomarkers of disease.

## Data Availability Statement

The raw data supporting the conclusions of this article will be made available by the authors, without undue reservation.

## Ethics Statement

The studies involving human participants were reviewed and approved by the Ethics Committee Shanghai Tongji Hospital. The patients/participants provided their written informed consent to participate in this study. Written informed consent was obtained from the individual(s) for the publication of any potentially identifiable images or data included in this article.

## Author Contributions

ND, CF, and RL collected the data and drafted and edited the manuscript. WZ, ML, and WX recruited the participants and contributed to the data collection. HT, PN, OT, IY, KO, MS, and SD provided the technical assistance with the machine learning aspects of the project. MS contributed to the manuscript draft and review. YL conceived and designed framework of this study, also funded and supervised this study, and reviewed the manuscript. All authors read and approved the final manuscript.

## Conflict of Interest

HT, PN, OT, IY, KO, SD, and MS are employees of Omniscient Neurotechnology, and they provided technical assistance with the machine learning aspects of the project. The remaining authors declare that the research was conducted in the absence of any commercial or financial relationships that could be construed as a potential conflict of interest.

## Publisher’s Note

All claims expressed in this article are solely those of the authors and do not necessarily represent those of their affiliated organizations, or those of the publisher, the editors and the reviewers. Any product that may be evaluated in this article, or claim that may be made by its manufacturer, is not guaranteed or endorsed by the publisher.

## References

[B1] Akiki T. J. Abdallah C. G. (2019). Determining the Hierarchical Architecture of the Human Brain Using Subject-Level Clustering of Functional Networks. *Sci. Rep.* 9:19290. 10.1038/s41598-019-55738-y 31848397 PMC6917755

[B2] Albert M. S. DeKosky S. T. Dickson D. Dubois B. Feldman H. H. Fox N. C. (2011). The diagnosis of mild cognitive impairment due to Alzheimer’s disease: recommendations from the National Institute on Aging-Alzheimer’s Association workgroups on diagnostic guidelines for Alzheimer’s disease. *Alzheimers Dement* 7 270–279. 10.1016/j.jalz.2011.03.008 21514249 PMC3312027

[B3] Almairac F. Herbet G. Moritz-Gasser S. de Champfleur N. M. Duffau H. (2015). The left inferior fronto-occipital fasciculus subserves language semantics: a multilevel lesion study. *Brain Struct. Funct.* 220 1983–1995. 10.1007/s00429-014-0773-1 24744151

[B4] Badhwar A. Tam A. Dansereau C. Orban P. Hoffstaedter F. Bellec P. (2017). Resting-state network dysfunction in Alzheimer’s disease: A systematic review and meta-analysis. *Alzheimers Dement* 8 73–85. 10.1016/j.dadm.2017.03.007 28560308 PMC5436069

[B5] Battista P. Salvatore C. Castiglioni I. (2017). Optimizing Neuropsychological Assessments for Cognitive, Behavioral, and Functional Impairment Classification: A Machine Learning Study. *Behav. Neurol.* 2017:1850909. 10.1155/2017/1850909 28255200 PMC5307249

[B6] Beam A. L. Manrai A. K. Ghassemi M. (2020). Challenges to the Reproducibility of Machine Learning Models in Health Care. *Jama* 323 305–306. 10.1001/jama.2019.20866 31904799 PMC7335677

[B7] Behzadi Y. Restom K. Liau J. Liu T. T. (2007). A component based noise correction method (CompCor) for BOLD and perfusion based fMRI. *Neuroimage* 37 90–101. 10.1016/j.neuroimage.2007.04.042 17560126 PMC2214855

[B8] Bondi M. W. Edmonds E. C. Jak A. J. Clark L. R. Delano-Wood L. McDonald C. R. (2014). Neuropsychological criteria for mild cognitive impairment improves diagnostic precision, biomarker associations, and progression rates. *J. Alzheimers Dis.* 42 275–289. 10.3233/jad-140276 24844687 PMC4133291

[B9] Braak H. Braak E. (1991). Neuropathological stageing of Alzheimer-related changes. *Acta Neuropathol.* 82 239–259. 10.1007/bf00308809 1759558

[B10] Braak H. Thal D. R. Ghebremedhin E. Del Tredici K. (2011). Stages of the pathologic process in Alzheimer disease: age categories from 1 to 100 years. *J. Neuropathol. Exp. Neurol.* 70 960–969. 10.1097/NEN.0b013e318232a379 22002422

[B11] Conner A. K. Briggs R. G. Sali G. Rahimi M. Baker C. M. Burks J. D. (2018). A Connectomic Atlas of the Human Cerebrum-Chapter 13: Tractographic Description of the Inferior Fronto-Occipital Fasciculus. *Oper. Neurosurg.* 15 S436–S443. 10.1093/ons/opy267 30260438 PMC6890527

[B12] Crowley R. J. Tan Y. J. Ioannidis J. P. A. (2020). Empirical assessment of bias in machine learning diagnostic test accuracy studies. *J. Am. Med. Inform. Assoc.* 27 1092–1101. 10.1093/jamia/ocaa075 32548642 PMC7647361

[B13] Davenport T. Kalakota R. (2019). The potential for artificial intelligence in healthcare. *Fut. Healthcare J.* 6 94–98. 10.7861/futurehosp.6-2-94 31363513 PMC6616181

[B14] Doyen S. Nicholas P. Poologaindran A. Crawford L. Young I. M. Romero-Garcia R. (2022). Connectivity-based parcellation of normal and anatomically distorted human cerebral cortex. *Hum. Brain Mapp.* 43 1358–1369. 10.1002/hbm.25728 34826179 PMC8837585

[B15] Doyen S. Taylor H. Nicholas P. Crawford L. Young I. Sughrue M. E. (2021). Hollow-tree super: a directional and scalable approach for feature importance in boosted tree models. *PLoS One* 16:e0258658. 10.1371/journal.pone.0258658 34695143 PMC8544862

[B16] Erk S. Spottke A. Meisen A. Wagner M. Walter H. Jessen F. (2011). Evidence of neuronal compensation during episodic memory in subjective memory impairment. *Arch. Gen. Psychiatry* 68 845–852. 10.1001/archgenpsychiatry.2011.80 21810648

[B17] Ewers M. Luan Y. Frontzkowski L. Neitzel J. Rubinski A. Dichgans M. (2021). Segregation of functional networks is associated with cognitive resilience in Alzheimer’s disease. *Brain* 144 2176–2185. 10.1093/brain/awab112 33725114 PMC8370409

[B18] Franzmeier N. Neitzel J. Rubinski A. Smith R. Strandberg O. Ossenkoppele R. (2020). Functional brain architecture is associated with the rate of tau accumulation in Alzheimer’s disease. *Nat. Commun.* 11:347. 10.1038/s41467-019-14159-1 31953405 PMC6969065

[B19] Franzmeier N. Rubinski A. Neitzel J. Kim Y. Damm A. Na D. L. (2019). Functional connectivity associated with tau levels in ageing, Alzheimer’s, and small vessel disease. *Brain* 142 1093–1107. 10.1093/brain/awz026 30770704 PMC6439332

[B20] Gill S. Mouches P. Hu S. Rajashekar D. MacMaster F. P. Smith E. E. (2020). Using Machine Learning to Predict Dementia from Neuropsychiatric Symptom and Neuroimaging Data. *J. Alzheimers Dis.* 75 277–288. 10.3233/jad-191169 32250302 PMC7306896

[B21] Giorgio J. Landau S. M. Jagust W. J. Tino P. Kourtzi Z. (2020). Modelling prognostic trajectories of cognitive decline due to Alzheimer’s disease. *Neuroimage Clin.* 26:102199. 10.1016/j.nicl.2020.102199 32106025 PMC7044529

[B22] Glasser M. F. Coalson T. S. Robinson E. C. Hacker C. D. Harwell J. Yacoub E. (2016). A multi-modal parcellation of human cerebral cortex. *Nature* 536 171–178. 10.1038/nature18933 27437579 PMC4990127

[B23] Grueso S. Viejo-Sobera R. (2021). Machine learning methods for predicting progression from mild cognitive impairment to Alzheimer’s disease dementia: a systematic review. *Alzheimers Res. Ther.* 13:162. 10.1186/s13195-021-00900-w 34583745 PMC8480074

[B24] Gupta A. Kahali B. (2020). Machine learning-based cognitive impairment classification with optimal combination of neuropsychological tests. *Alzheimers Dement* 6:e12049. 10.1002/trc2.12049 32699817 PMC7369403

[B25] Huang K. Lin Y. Yang L. Wang Y. Cai S. Pang L. (2020). A multipredictor model to predict the conversion of mild cognitive impairment to Alzheimer’s disease by using a predictive nomogram. *Neuropsychopharmacology* 45 358–366. 10.1038/s41386-019-0551-0 31634898 PMC6901533

[B26] Isensee F. Schell M. Pflueger I. Brugnara G. Bonekamp D. Neuberger U. (2019). Automated brain extraction of multisequence MRI using artificial neural networks. *Hum. Brain Mapp.* 40 4952–4964. 10.1002/hbm.24750 31403237 PMC6865732

[B27] Jitsuishi T. Yamaguchi A. (2022). Searching for optimal machine learning model to classify mild cognitive impairment (MCI) subtypes using multimodal MRI data. *Sci. Rep.* 12:4284. 10.1038/s41598-022-08231-y 35277565 PMC8917197

[B28] Jo T. Nho K. Saykin A. J. (2019). Deep Learning in Alzheimer’s Disease: Diagnostic Classification and Prognostic Prediction Using Neuroimaging Data. *Front. Aging Neurosci.* 11:220. 10.3389/fnagi.2019.00220 31481890 PMC6710444

[B29] Kumar S. Oh I. Schindler S. Lai A. M. Payne P. R. O. Gupta A. (2021). Machine learning for modeling the progression of Alzheimer disease dementia using clinical data: a systematic literature review. *JAMIA Open* 4:ooab052. 10.1093/jamiaopen/ooab052 34350389 PMC8327375

[B30] Kumar S. Zomorrodi R. Ghazala Z. Goodman M. S. Blumberger D. M. Cheam A. (2017). Extent of Dorsolateral Prefrontal Cortex Plasticity and Its Association With Working Memory in Patients With Alzheimer Disease. *JAMA Psychiatry* 74 1266–1274. 10.1001/jamapsychiatry.2017.3292 29071355 PMC6583382

[B31] Liang P. Wang Z. Yang Y. Jia X. Li K. (2011). Functional disconnection and compensation in mild cognitive impairment: evidence from DLPFC connectivity using resting-state fMRI. *PLoS One* 6:e22153. 10.1371/journal.pone.0022153 21811568 PMC3141010

[B32] Liao D. A. Kronemer S. I. Yau J. M. Desmond J. E. Marvel C. L. (2014). Motor system contributions to verbal and non-verbal working memory. *Front. Hum. Neurosci.* 8:753. 10.3389/fnhum.2014.00753 25309402 PMC4173669

[B33] Lo T. W. B. Karameh W. K. Barfett J. J. Fornazzari L. R. Munoz D. G. Schweizer T. A. (2020). Association Between Neuropsychiatric Symptom Trajectory and Conversion to Alzheimer Disease. *Alzheimer Dis. Assoc. Disord.* 34 141–147. 10.1097/wad.0000000000000356 31633557

[B34] Marder K. (2005). Vitamin E and donepezil for the treatment of mild cognitive impairment. *Curr. Neurol. Neurosci. Rep.* 5 337–338. 10.1007/s11910-005-0056-6 16131415

[B35] Marvel C. L. Desmond J. E. (2012). From storage to manipulation: how the neural correlates of verbal working memory reflect varying demands on inner speech. *Brain Lang.* 120 42–51. 10.1016/j.bandl.2011.08.005 21889195 PMC3242899

[B36] Moritz-Gasser S. Herbet G. Duffau H. (2013). Mapping the connectivity underlying multimodal (verbal and non-verbal) semantic processing: a brain electrostimulation study. *Neuropsychologia* 51 1814–1822. 10.1016/j.neuropsychologia.2013.06.007 23778263

[B37] Odusami M. Maskeliūnas R. Damaševičius R. Krilavičius T. (2021). Analysis of Features of Alzheimer’s Disease: Detection of Early Stage from Functional Brain Changes in Magnetic Resonance Images Using a Finetuned ResNet18 Network. *Diagnostics* 11:1071. 10.3390/diagnostics11061071 34200832 PMC8230447

[B38] Omniscient Neurotechnology (2020). *Infinitome.* Available online at: https://infinitome.o8t.com/ (accessed September 14, 2021).

[B39] Panch T. Szolovits P. Atun R. (2018). Artificial intelligence, machine learning and health systems. *J. Glob. Health* 8:020303. 10.7189/jogh.08.020303 30405904 PMC6199467

[B40] Paulesu E. Frith C. D. Frackowiak R. S. (1993). The neural correlates of the verbal component of working memory. *Nature* 362 342–345. 10.1038/362342a0 8455719

[B41] Prasad G. Nir T. M. Toga A. W. Thompson P. M. (2013). TRACTOGRAPHY DENSITY AND NETWORK MEASURES IN ALZHEIMER’S DISEASE. *Proc. IEEE Int. Sympos. Biomed. Imaging* 2013 692–695. 10.1109/isbi.2013.6556569 25404994 PMC4232938

[B42] Prince M. Bryce R. Albanese E. Wimo A. Ribeiro W. Ferri C. P. (2013). The global prevalence of dementia: a systematic review and metaanalysis. *Alzheimers Dement* 9 63–75.e2. 10.1016/j.jalz.2012.11.007 23305823

[B43] Roelofs A. Piai V. (2011). Attention demands of spoken word planning: a review. *Front. Psychol.* 2:307. 10.3389/fpsyg.2011.00307 22069393 PMC3209602

[B44] Shi J. Liu B. (2020). Stage detection of mild cognitive impairment via fMRI using Hilbert Huang transform based classification framework. *Med. Phys.* 47 2902–2915. 10.1002/mp.14183 32302413

[B45] Sisi W. Yixuan K. (2018). The causal role of right dorsolateral prefrontal cortex in visual working memory. *Acta Psychol. Sin.* 50 727–738. 10.3724/SP.J.1041.2018.00727

[B46] Stamate D. Smith R. Tsygancov R. Vorobev R. Langham J. Stahl D. (2020). “Applying Deep Learning to Predicting Dementia and Mild Cognitive Impairment,” in *Artificial Intelligence Applications and Innovations*, eds Maglogiannis I. Iliadis L. Pimenidis E. (Cham: Springer International Publishing), 308–319.

[B47] Syaifullah A. H. Shiino A. Kitahara H. Ito R. Ishida M. Tanigaki K. (2020). Machine Learning for Diagnosis of AD and Prediction of MCI Progression From Brain MRI Using Brain Anatomical Analysis Using Diffeomorphic Deformation. *Front. Neurol.* 11:576029. 10.3389/fneur.2020.576029 33613411 PMC7893082

[B48] Tao W. Sun J. Li X. Shao W. Pei J. Yang C. (2020). The Anterior-posterior Functional Connectivity Disconnection in the Elderly with Subjective Memory Impairment and Amnestic Mild Cognitive Impairment. *Curr. Alzheimer Res.* 17 373–381. 10.2174/1567205017666200525015017 32448103

[B49] Topiwala A. Suri S. Allan C. Valkanova V. Filippini N. Sexton C. E. (2019). Predicting cognitive resilience from midlife lifestyle and multi-modal MRI: a 30-year prospective cohort study. *PLoS One* 14:e0211273. 10.1371/journal.pone.0211273 30779761 PMC6380585

[B50] van Loenhoud A. C. van der Flier W. M. Wink A. M. Dicks E. Groot C. Twisk J. (2019). Cognitive reserve and clinical progression in Alzheimer disease: a paradoxical relationship. *Neurology* 93 e334–e346. 10.1212/wnl.0000000000007821 31266904 PMC6669930

[B51] Vega J. N. Newhouse P. A. (2014). Mild cognitive impairment: diagnosis, longitudinal course, and emerging treatments. *Curr. Psychiatry Rep.* 16:490. 10.1007/s11920-014-0490-8 25160795 PMC4169219

[B52] Villemagne V. L. Burnham S. Bourgeat P. Brown B. Ellis K. A. Salvado O. (2013). Amyloid β deposition, neurodegeneration, and cognitive decline in sporadic Alzheimer’s disease: a prospective cohort study. *Lancet Neurol.* 12 357–367. 10.1016/s1474-4422(13)70044-9 23477989

[B53] Vogel J. W. Young A. L. Oxtoby N. P. Smith R. Ossenkoppele R. Strandberg O. T. (2021). Four distinct trajectories of tau deposition identified in Alzheimer’s disease. *Nat. Med.* 27 871–881. 10.1038/s41591-021-01309-6 33927414 PMC8686688

[B54] Wang K. Liang M. Wang L. Tian L. Zhang X. Li K. (2007). Altered functional connectivity in early Alzheimer’s disease: a resting-state fMRI study. *Hum. Brain Mapp.* 28 967–978. 10.1002/hbm.20324 17133390 PMC6871392

[B55] Wang Z. Qiao K. Chen G. Sui D. Dong H. M. Wang Y. S. (2019). Functional Connectivity Changes Across the Spectrum of Subjective Cognitive Decline, Amnestic Mild Cognitive Impairment and Alzheimer’s Disease. *Front. Neuroinform.* 13:26. 10.3389/fninf.2019.00026 31105548 PMC6491896

[B56] Wee C. Y. Yap P. T. Zhang D. Denny K. Browndyke J. N. Potter G. G. (2012). Identification of MCI individuals using structural and functional connectivity networks. *Neuroimage* 59 2045–2056. 10.1016/j.neuroimage.2011.10.015 22019883 PMC3254811

[B57] Weiler M. Casseb R. F. de Campos B. M. de Ligo Teixeira C. V. Carletti-Cassani A. F. M. K. Vicentini J. E. (2018). Cognitive Reserve Relates to Functional Network Efficiency in Alzheimer’s Disease. *Front. Aging Neurosci.* 10:255. 10.3389/fnagi.2018.00255 30186154 PMC6111617

[B58] Weiler M. Stieger K. C. Long J. M. Rapp P. R. (2020). Transcranial Magnetic Stimulation in Alzheimer’s Disease: Are We Ready?. *eNeuro* 7:ENEURO.0235-19.2019, 10.1523/eneuro.0235-19.2019 31848209 PMC6948923

[B59] Wisch J. K. Roe C. M. Babulal G. M. Schindler S. E. Fagan A. M. Benzinger T. L. (2020). Resting State Functional Connectivity Signature Differentiates Cognitively Normal from Individuals Who Convert to Symptomatic Alzheimer’s Disease. *J. Alzheimers Dis.* 74 1085–1095. 10.3233/jad-191039 32144983 PMC7183885

[B60] Ye C. Mori S. Chan P. Ma T. (2019). Connectome-wide network analysis of white matter connectivity in Alzheimer’s disease. *Neuroimage Clin.* 22:101690. 10.1016/j.nicl.2019.101690 30825712 PMC6396432

[B61] Yeo B. T. Krienen F. M. Sepulcre J. Sabuncu M. R. Lashkari D. Hollinshead M. (2011). The organization of the human cerebral cortex estimated by intrinsic functional connectivity. *J. Neurophysiol.* 106 1125–1165. 10.1152/jn.00338.2011 21653723 PMC3174820

[B62] Yordanova Y. N. Duffau H. Herbet G. (2017). Neural pathways subserving face-based mentalizing. *Brain Struct. Funct.* 222 3087–3105. 10.1007/s00429-017-1388-0 28243761

