# Supplementary material for: Machine Learning Decomposition of the Anatomy of Neuropsychological Deficit in Alzheimer’s Disease and Mild Cognitive Impairment
Source: Front Aging Neurosci. 2022 May 3;14:854733. doi: 10.3389/fnagi.2022.854733 (PMC9110794; doi:10.3389/fnagi.2022.854733)
Supplement: Supplementary file 1 [file Data_Sheet_1.docx]

Supplementary Material

## Supplementary Figures


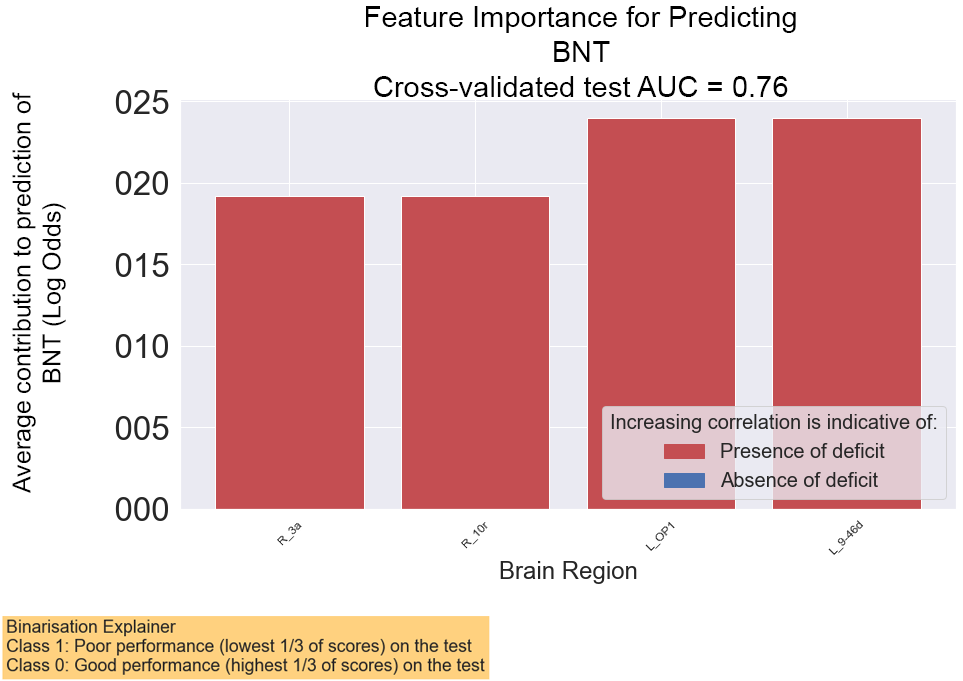
**Supplementary Figure 1.** Parcels associated with deficit and absence of deficit in the Boston Naming Test, along with their log odds based on a tertile split of scores.


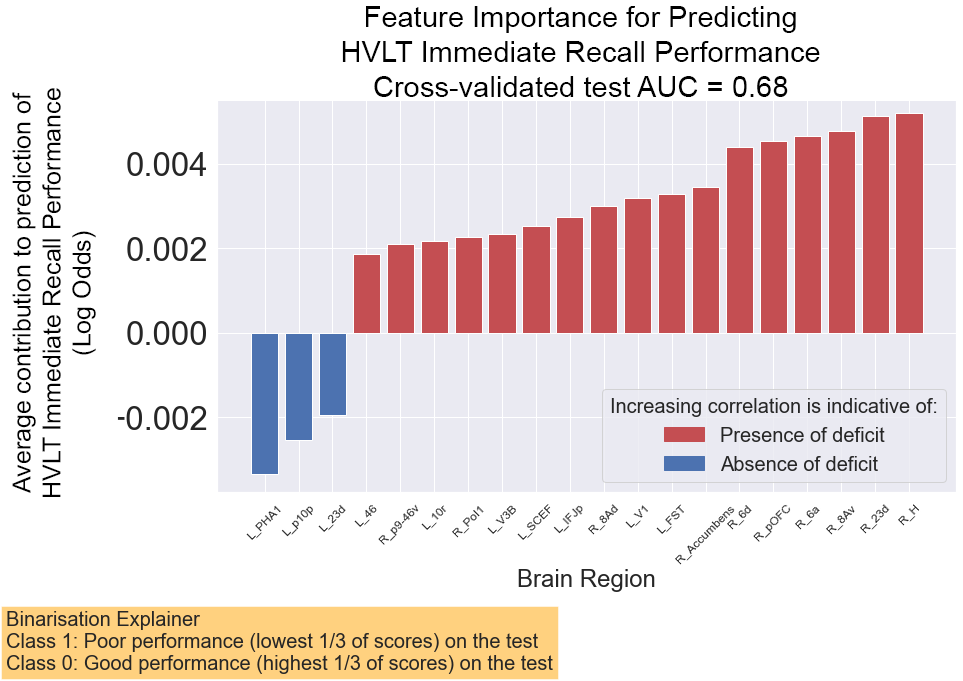


**Supplementary Figure 2.** Parcels associated with deficit and absence of deficit in the Hopkins Verbal Learning Test Immediate Memory, along with their log odds based on a tertile split of scores.


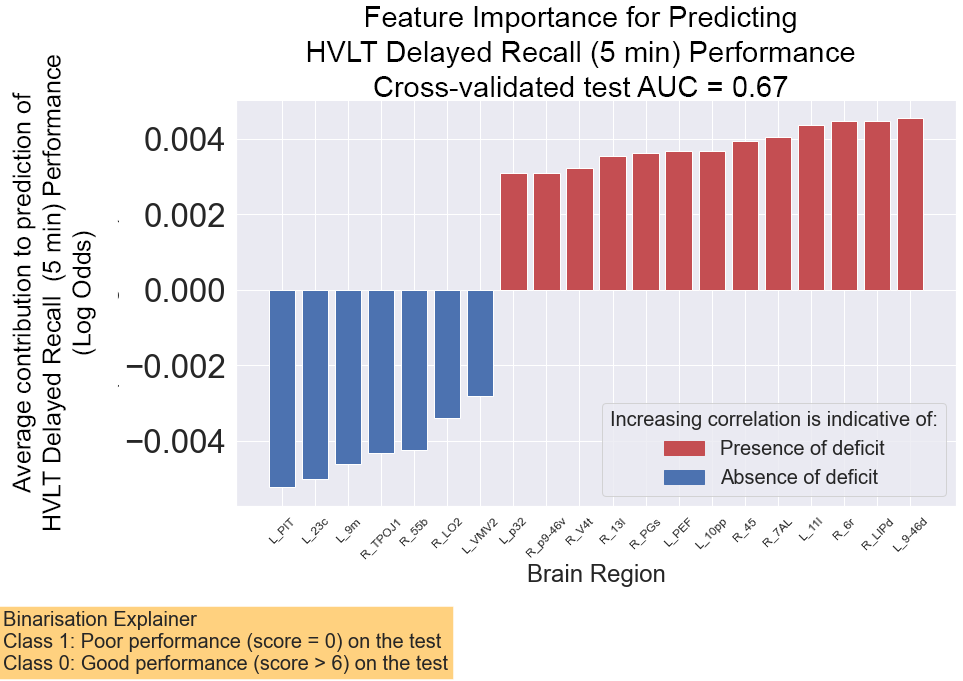


**Supplementary Figure 3.** Parcels associated with deficit and absence of deficit in the Hopkins Verbal Learning Test Delayed Recall (5 min), along with their log odds.


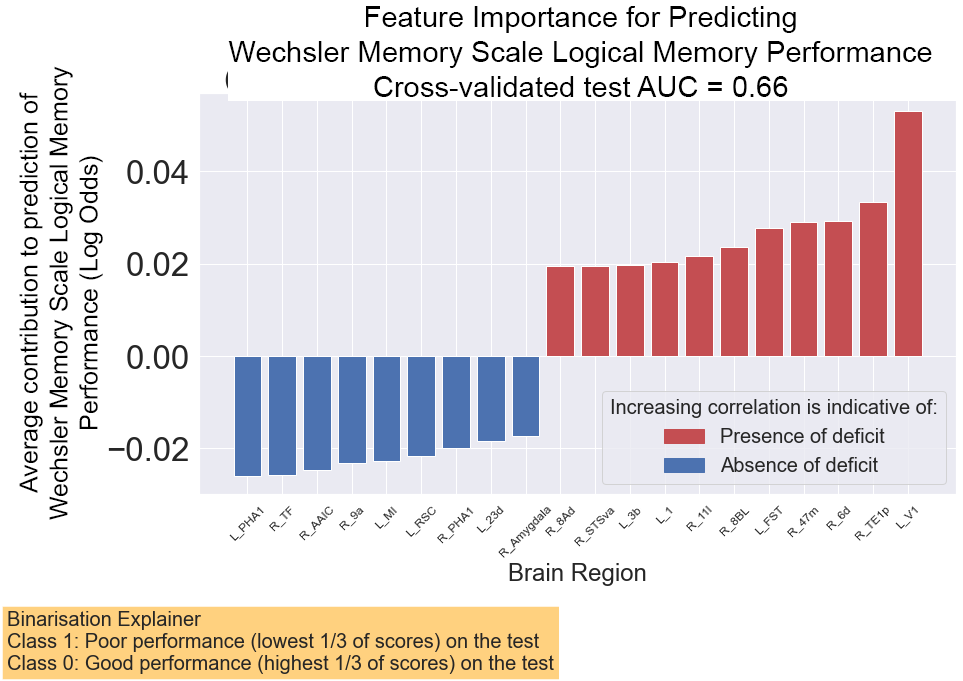


**Supplementary Figure 4.** Parcels associated with deficit and absence of deficit in the Wechsler Memory Scale Logical Memory, along with their log odds, based on a tertile split of scores.


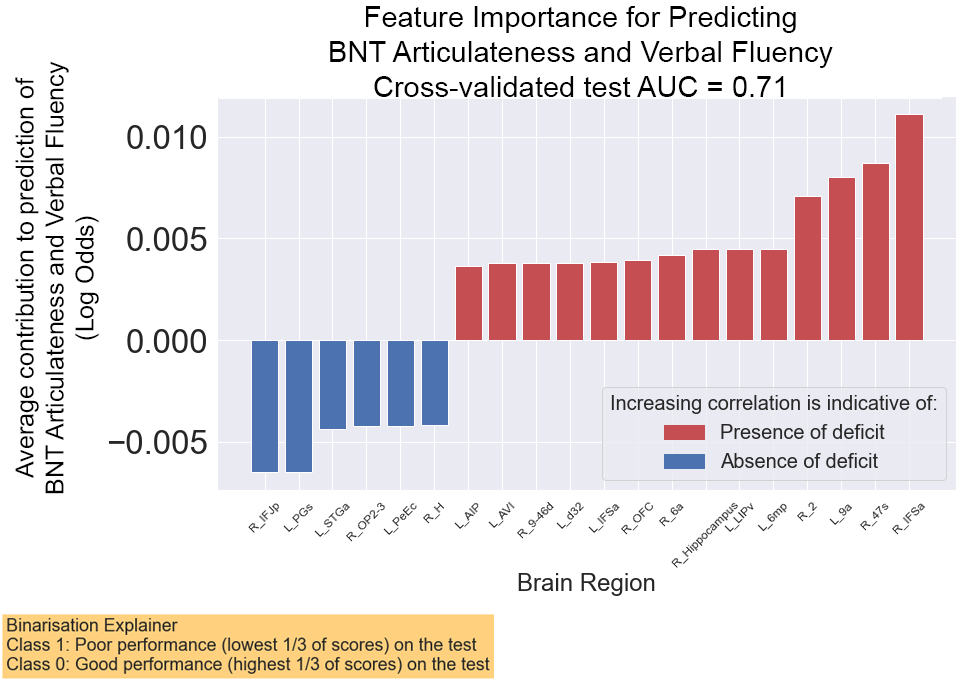
**Supplementary Figure 5.** Parcels associated with deficit and absence of deficit in the articulateness and fluency part of the Boston Naming Test, along with their log odds, based on a tertile split of scores.

**
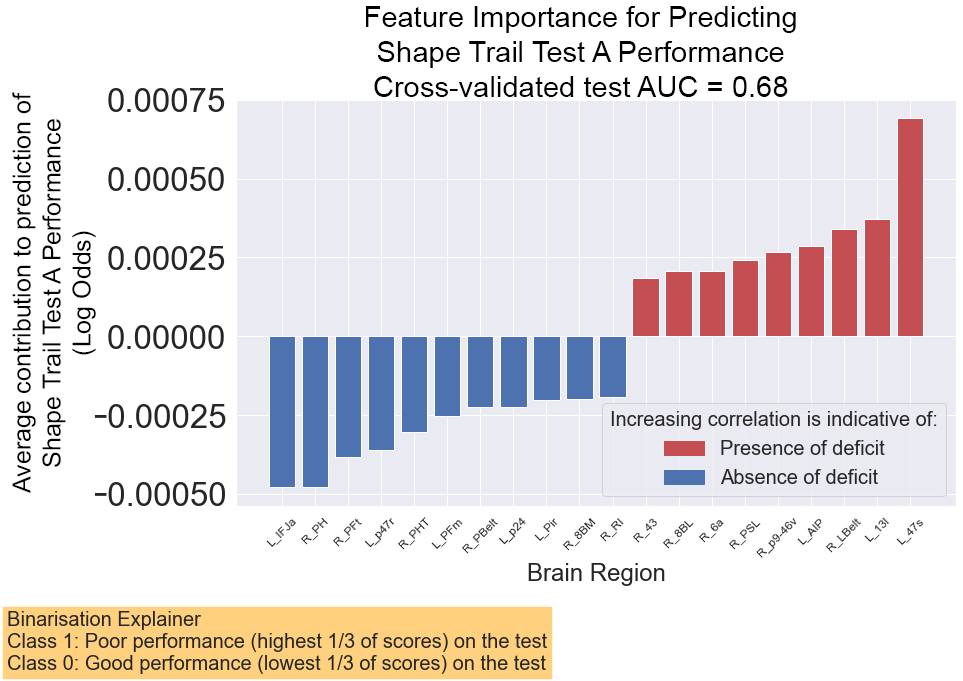
Supplementary Figure 6.** Parcels associated with deficit and absence of deficit in the Shape Trail Test Part A, along with their log odds, based on a tertile split of scores.


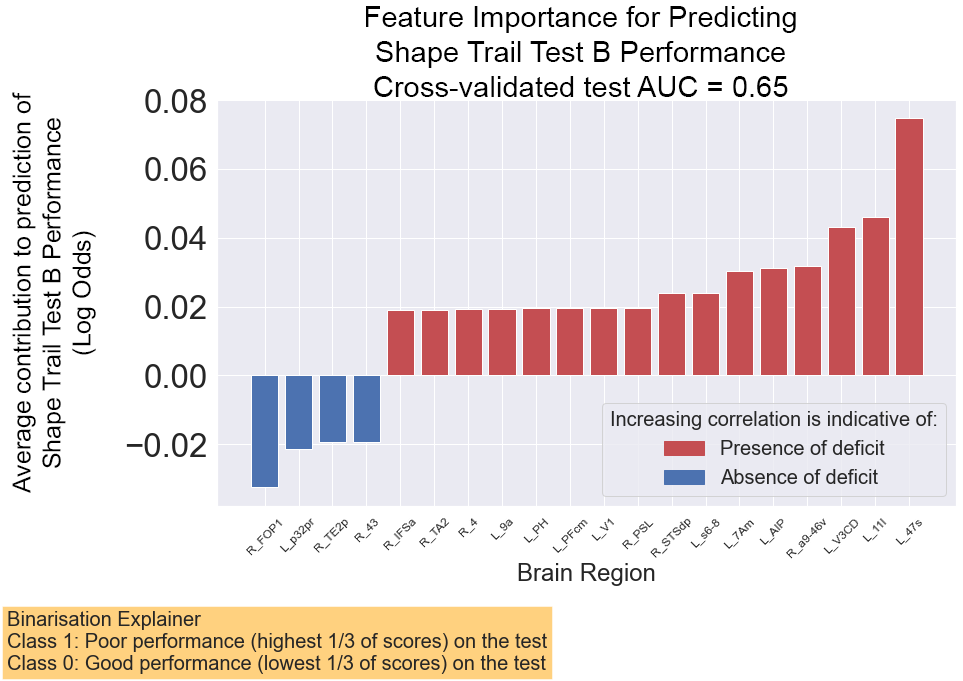
**Supplementary Figure 7.** Parcels associated with deficit and absence of deficit in the Shape Trail Test Part B, along with their log odds, based on a tertile split of scores.


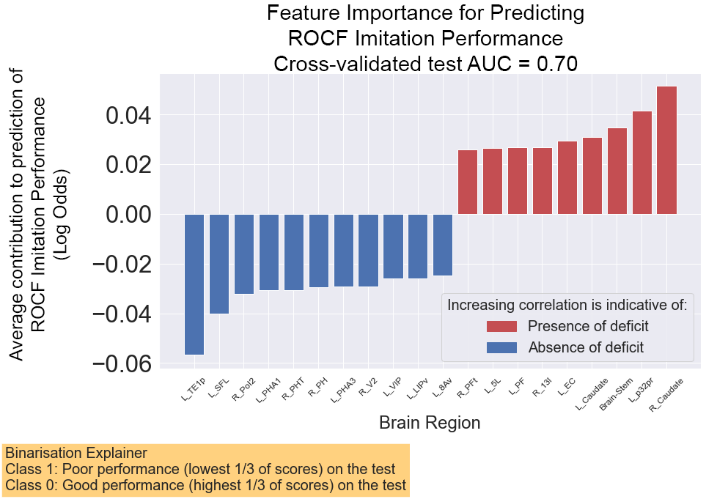


**Supplementary Figure 8.** Parcels associated with deficit and absence of deficit in the Rey Osterrieth Complex Figure Test Imitation, along with their log odds, based on a tertile split of scores.

**
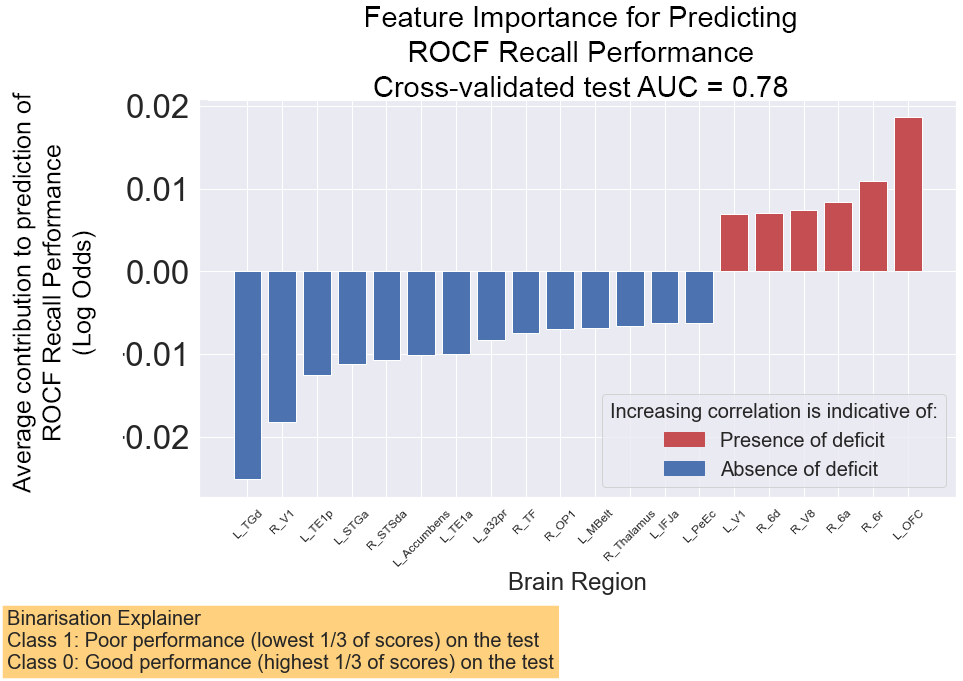
Supplementary Figure 9.** Parcels associated with deficit and absence of deficit in the Rey Osterrieth Complex Figure Test Recall, along with their log odds, based on a tertile split of scores.

**
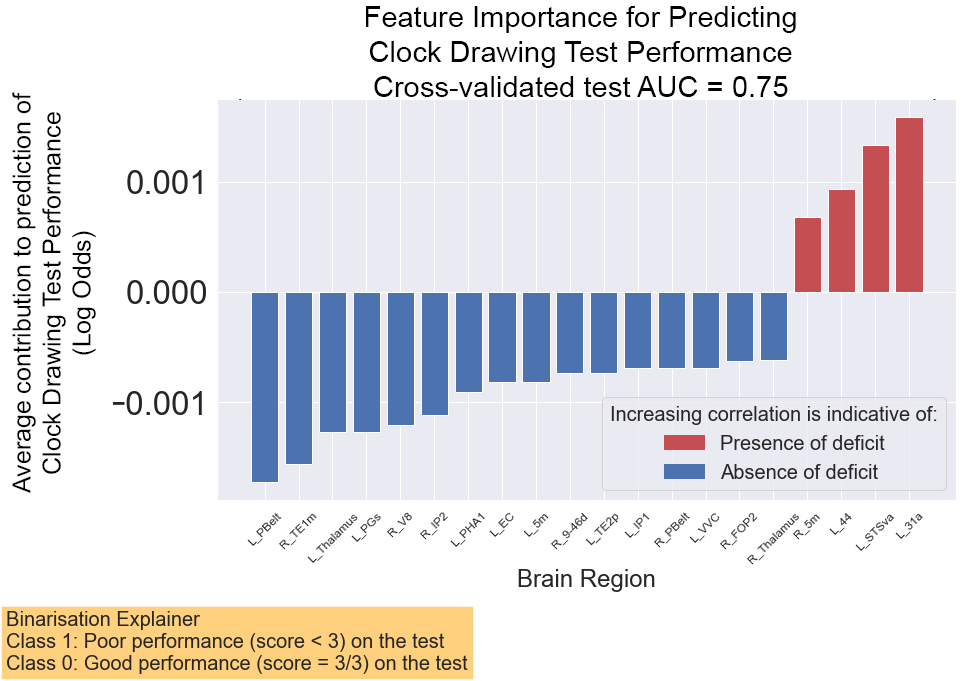
Supplementary Figure 10.** Parcels associated with deficit and absence of deficit in the Clock Drawing Test, along with their log odds.


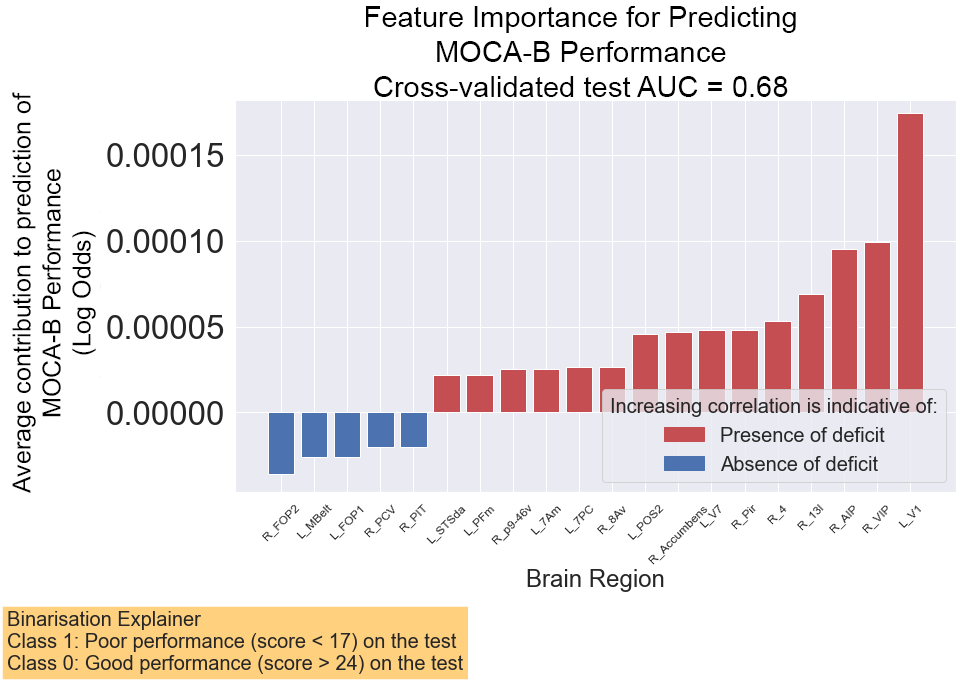
**Supplementary Figure 11.** Parcels associated with deficit and absence of deficit in the MOCA-B, along with their log odds.
